# Automated Vision-Based Sensing of Paediatric Pain Using 2D-Facial Landmark Trajectories Derived from 3D Geometric Normalisation and a Spatial-Temporal Attention Long Short-Term Memory (STA-LSTM) Network

**DOI:** 10.3390/s26185823

**Published:** 2026-09-14

**Authors:** Teddy Fabila, Tiehua Du, Chin Wen Tan, Rehena Sultana, Choon Looi Bong, Ban Leong Sng

**Affiliations:** 1 Department of Paediatric Anaesthesia, KK Women’s and Children’s Hospital, Singapore 229899, Singapore; teddy.fabila@kkh.com.sg; 2 Biomedical Engineering and Materials Group, Nanyang Polytechnic, Singapore 569830, Singapore; du_tiehua@nyp.edu.sg; 3 Department of Women’s Anaesthesia, KK Women’s and Children’s Hospital, Singapore 229899, Singapore; tan.chin.wen@kkh.com.sg (C.W.T.); rehena.sultana@duke-nus.edu.sg (R.S.); sng.ban.leong@singhealth.com.sg (B.L.S.); 4 Duke-NUS Medical School, Singapore 169857, Singapore

**Keywords:** pain assessment, paediatric pain, facial landmark analysis, computer vision, MediaPipe Face Mesh, spatial-temporal attention, long short-term memory, vision-based sensing

## Abstract

**Highlights:**

**What are the main findings?**

- A vision-based sensing pipeline using 2D trajectories derived from 3D-normalised landmarks and a STA-LSTM network can accurately distinguish observer-labelled pain from no-pain states in children (AUC 0.994, accuracy 97.15%)
- The system operates on landmark coordinates rather than raw facial-image pixels, reducing reliance on facial texture during model inference, demonstrating high-performance automated pain detection in a real-world perioperative cohort of 125 children.

**What are the implications of the main findings?**

- Facial landmark trajectories provide a practical, privacy-conscious signal representation for non-contact, continuous monitoring of paediatric pain at the bedside.
- This validated sensing framework offers a foundation for developing integrated, multimodal sensor systems and clinical decision-support tools to improve pain recognition in children.

**Abstract:**

**Background:** Accurate pain assessment in children remains challenging because conventional behavioural assessment tools are subjective and provide only intermittent observations. This study evaluated a vision-based sensing system for automated paediatric pain detection using two-dimensional facial landmark trajectories derived from three-dimensional geometric normalisation of frontal facial video recordings. **Methods:** In a prospective observational study, 125 children aged 6–17 years undergoing elective surgery had frontal facial videos recorded pre- and postoperatively using a custom-developed iOS mobile application. Video frames were processed using the MediaPipe Face Mesh framework to estimate 478 facial landmarks with three-dimensional coordinates. After three-dimensional normalisation, the normalised *x*- and *y*-coordinates were segmented into one-second trajectories, and analysed using a previously developed Spatial-Temporal Attention Long Short-Term Memory (STA-LSTM) framework. Reference pain labels (pain vs. no pain) were derived from observer-rated revised Face, Legs, Activity, Cry, Consolability (r-FLACC) scores. **Results:** The sensing system analysed 971 annotated clips and was trained on 6438 balanced landmark trajectories. On an independent validation dataset, it achieved an area under the receiver operating characteristic curve of 0.994, with an accuracy of 97.15%, precision of 86.78%, recall of 97.83%, and F1-score of 91.97% for binary pain classification. **Conclusions:** A landmark-based, non-contact vision sensor combined with a STA-LSTM network achieved high performance for automated detection of observer-labelled pain states in children, supporting facial landmark trajectories as a practical signal representation for privacy-conscious continuous pain monitoring.

## 1. Introduction

Continuous and accurate pain assessment in children is fundamental for safe perioperative care, yet remains difficult in routine practice. Conventional methods rely on self-report or observer-based behavioural scales such as the revised Face, Legs, Activity, Cry, Consolability (r-FLACC) scale, which are subjective and are only administered intermittently by trained staff rather than continuously at the bedside [1,2,3]. These constraints limit timely recognition of pain and hinder closed-loop or decision-support systems for paediatric analgesia, driving interest in automated, non-contact approaches that may provide reproducible behavioral signals to support pain assessment.

Vision-based sensing offers a non-contact way to capture facial behaviour related to pain and to derive objective behavioural signals over time. Recent advances in artificial intelligence (AI) and computer vision have shown that automated analysis of facial appearance from video recordings can discriminate pain from no-pain states using deep learning architectures [4,5], including convolutional neural networks, recurrent neural networks, or transformer-based architectures [4,5,6,7,8]. However, image-based methods remain sensitive to illumination, viewing angle, facial occlusion, and background variation. In addition, image-based analysis requires processing identifiable facial images, which poses privacy and data governance challenges, particularly in paediatric populations [4,5].

Facial landmark analysis offers an alternative representation in which facial behaviour is encoded as geometric movement trajectories rather than raw facial appearance. MediaPipe Face Mesh estimates 478 three-dimensional facial landmarks from monocular video, providing a compact representation of facial geometry and motion while reducing direct dependence on facial texture [9]. Landmark data nevertheless remain derived from an individual’s face and therefore continue to require appropriate privacy and governance safeguards. Because pain expression is dynamic, analysis of isolated landmarks or individual video frames may not adequately represent the temporal evolution of facial behaviour [10,11,12].

The Spatial-Temporal Attention Long Short-Term Memory Network (STA-LSTM) combines long short-term memory modelling of sequential data with attention mechanisms that identify informative spatial landmark features and temporal segments. Tan et al. previously developed and evaluated a customised STA-LSTM for automated facial pain detection in adults. Their system used 478 facial keypoints extracted from short facial-video sequences to model changes in facial geometry across both spatial and temporal domains and classify adult pain intensity [12]. This established an adult landmark-based pain-sensing framework but did not determine whether the same representation and temporal-learning strategy would remain effective in children.

Paediatric application represents a distinct domain-transfer problem because facial morphology, developmental patterns of pain expression, behaviour during assessment, and the perioperative context differ from those of adults. Existing paediatric computer-vision studies have predominantly examined neonatal or infant populations or have relied on appearance-based facial images rather than normalised three-dimensional landmark trajectories [5,6,7,8]. The feasibility of applying the established adult STA-LSTM framework to school-aged children and adolescents remains uncertain.

This study evaluates the feasibility of transferring the established Tan et al. STA-LSTM framework to paediatric perioperative pain detection. Using normalised three-dimensional facial landmark trajectories rather than raw images, it assesses whether an adult-trained spatial-temporal model can distinguish observer-labelled pain from no-pain states in children. This work provides the first prospective evaluation of a landmark-based STA-LSTM pain-sensing framework in school-aged and adolescent children, using a privacy-conscious geometric representation rather than raw facial images.

## 2. Materials and Methods

### 2.1. Study Design and Vision-Based Sensing Pipeline

This prospective observational study was conducted at KK Women’s and Children’s Hospital (KKH), Singapore, between July 2022 and March 2024 following approval from the SingHealth Centralised Institutional Review Board (CIRB Ref. No. 2019/2293). The study was prospectively registered at ClinicalTrials.gov (NCT04011189) and reported in accordance with the Transparent Reporting of a Multivariable Prediction Model for Individual Prognosis or Diagnosis (TRIPOD) recommendations for prediction model studies [13]. Written informed consent was obtained from parents or legal guardians, with assent obtained from children where appropriate.

**Figure 1:**
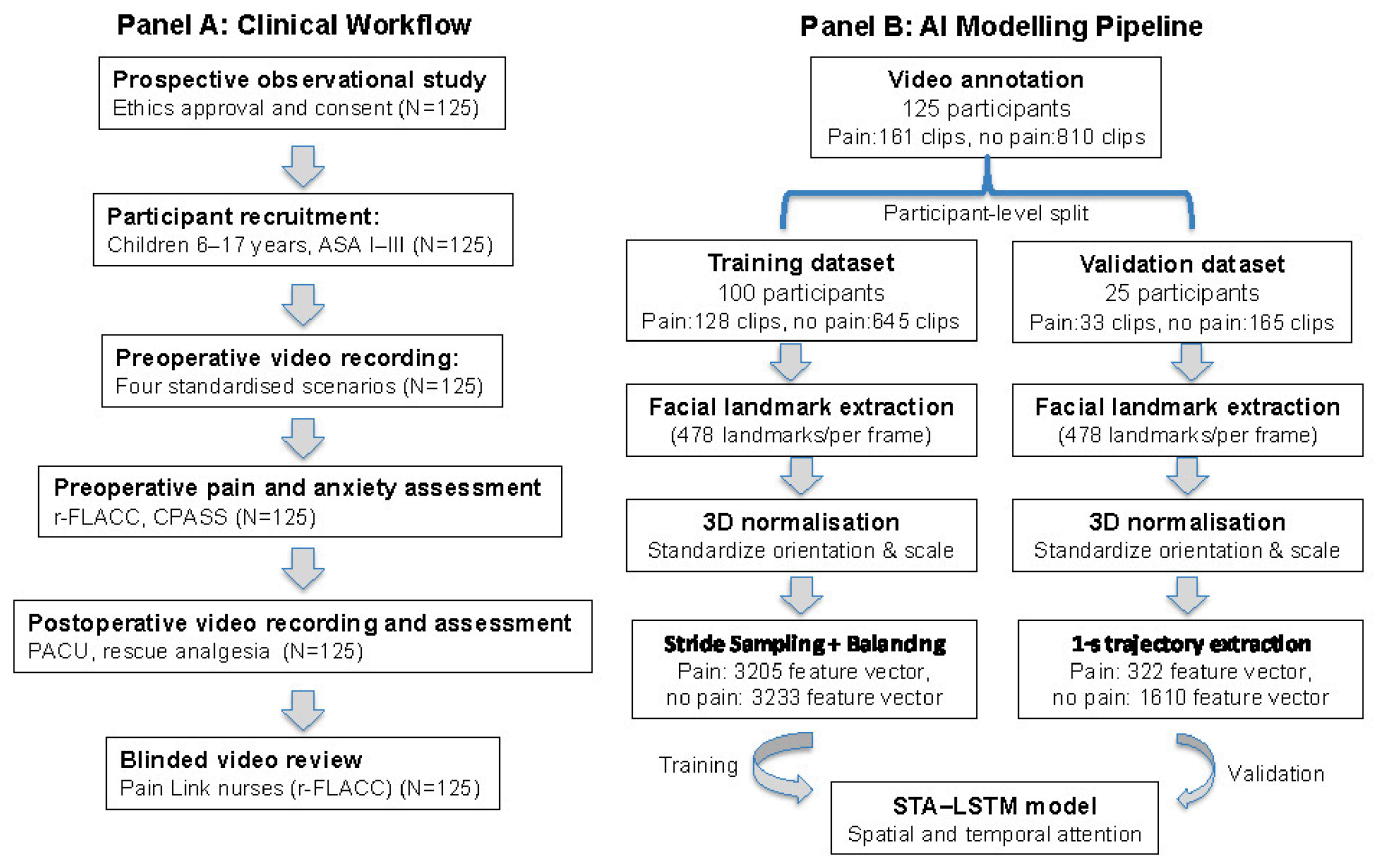
Clinical workflow and AI modelling pipeline for paediatric pain detection using perioperative video analysis and a spatial-temporal attention long short-term memory (STA–LSTM) model. ASA: American Society of Anaesthesiologists physical status, NRS: Numeric Rating Scale, r-FLACC: Revised Face, Legs, Activity, Cry, Consolability scale, CPASS: Children’s Pain Anxiety Symptoms Scale, PACU: Post-anaesthesia Care Unit.

The study evaluated the applicability of a previously published facial landmark-based Spatial-Temporal Attention Long Short-Term Memory (STA-LSTM) framework for automated paediatric pain detection as part of a five-stage sequential sensing pipeline, as follows: (1) frontal video acquisition using an iOS mobile application, (2) facial landmark localisation, (3) three-dimensional geometric normalisation, (4) temporal feature learning using the STA-LSTM framework, and (5) binary pain classification (pain vs. no pain) (Figure 1).

Children aged 6–17 years with American Society of Anesthesiologists (ASA) physical status I–III scheduled for elective surgery under general anaesthesia were prospectively recruited. Exclusion criteria were conditions likely to alter behavioural facial responses independently of acute pain (global developmental delay, significant cognitive impairment, autism spectrum disorder, attention deficit hyperactivity disorder, chronic pain syndromes, pharmacological premedication for severe anxiety, and previous traumatic pain experiences).

### 2.2. Vision-Based Signal Acquisition

Eligible children were recruited in the hospital’s reception area. Following enrolment, frontal-view video recordings capturing facial expression and body posture were obtained using a customised iOS mobile phone application. Each recording session lasted approximately 10 to 20 min and comprised four standardised scenarios, as follows: sitting, sit-to-stand, standing, and walking. Videos were captured in high-definition resolution (2160 × 3840 pixels) in MPEG-4 format with H.264 encoding at 30 frames per second. A second frontal-view video recording was performed postoperatively using the same protocol. Raw video files were stored temporarily on the mobile device with access restricted to authorised study personnel. No patient-identifiable information was stored on the recording device.

### 2.3. Reference Pain Labels

The r-FLACC scale was used as a consistent observer-based measure across all age groups and to align pain labels with behaviours visible in the recordings. Although self-report may be preferred in children who can communicate, it was not used as the primary reference because the model was trained to identify observable pain-related behaviour. The r-FLACC has established validity and reliability and permits individualised behavioural descriptors [3,14].

Pain was assessed during the four standardised scenarios by the responsible anaesthesia nurse and a trained clinician. The same clinician and a PACU nurse repeated the assessments postoperatively before discharge. Preoperative pain-related anxiety was measured using the Children’s Pain Anxiety Symptoms Scale (CPASS) as an exploratory measure of baseline pain-related anxiety [15]. Rescue analgesia administered in the PACU was recorded.

Six Pain Link Nurses with training and experience in paediatric pain assessment reviewed the videos while blinded to clinical information and preoperative scores. Each scenario was independently scored by two Pain Link Nurses using r-FLACC to assess inter-rater reliability. For binary classification, an r-FLACC score of 0 was labelled as no pain and a score >0 as pain, representing the presence of any observed pain-related behaviour. If there was a difference of 2 or greater between the independent reviewers, a third clinician (senior anesthesiologist) would review the video and the discrepancy would be resolved by consensus.

These observer-derived labels were used solely as reference targets for supervised learning and were not included in the feature extraction pipeline or the model’s prediction inputs.

### 2.4. Dataset Construction and Class Balancing

Continuous video recordings were processed offline and segmented into one-second clips, each comprising 30 consecutive video frames. A one-second temporal window was selected to preserve short-duration facial dynamics while maintaining computational efficiency for temporal sequence modelling. The original dataset exhibited class imbalance, with pain clips less frequent than no-pain clips. To minimise classifier bias, class balancing was applied only to the training dataset, as follows: pain clips were oversampled by using overlapping temporal windows with smaller strides, whereas no-pain clips were undersampled with larger strides. The independent validation dataset remained unchanged and retained the original clinical class distribution to provide an unbiased estimate of model performance.

### 2.5. Facial Landmark Extraction and Three-Dimensional Geometric Normalisation

Rather than using facial image pixels and model inputs, the proposed framework represented facial motion as landmark trajectories. Each frame of the recorded videos was processed using the MediaPipe Face Mesh (version 0.10.11; Google LLC, Mountain View, CA, USA) framework to estimate 478 facial landmarks from a monocular image [9]. Only the extracted landmark coordinates were used for subsequent processing and model training; the recorded video frames were not used directly during model training or inference.

Each landmark was represented by Cartesian coordinates

$$p_{i}=(x\_i,y\_i,z\_i),i=1,\ldots ,478$$

where x_i and y_i denote image-plane coordinates and z_i represents the relative depth estimated by MediaPipe. The facial geometry for each frame was represented as

$$P=\{p_{1},p_{2},\ldots ,p_{478}\}$$

Compared with pixel-based image representations, landmark coordinates provide a compact geometric representation of facial motion while substantially reducing input dimensionality. Because only geometric information is retained, identifiable facial texture is excluded from subsequent analysis.

To minimise variability from head movement, facial orientation, camera distance, and scale differences, all landmark sequences underwent rigid three-dimensional geometric normalisation before temporal analysis. Landmark coordinates were first translated to the facial centroid

$$P^{\prime }=P-c,$$

where

$$c=\frac{1}{N}\sum_{i=1}^{N}p_{i}$$

represents the centroid of all detected facial landmarks.

Scale normalisation was subsequently performed according to

$$P^{\prime \prime }=\frac{P^{\prime }}{s},$$

where s denotes the facial scaling factor estimated from stable anatomical landmarks. Rotational alignment was then performed using an orthogonal Procrustes transformation solved by Singular Value Decomposition (SVD), minimising the least-squares distance between corresponding landmark configurations while preserving rigid facial geometry [16,17]. After normalisation, landmark trajectories primarily represented facial deformation associated with behavioural expression rather than head pose or camera placement.

After 3D normalisation, the facial landmarks are transformed into a standardised, approximately frontal-facing representation. Consequently, much of the depth variation associated with head pose has already been corrected, making the z-coordinate less informative for the subsequent pain classification task. We therefore discarded the normalised z-coordinate and use only the normalised x and y coordinates as the model input, resulting in 956 features per frame (478 landmarks × 2 coordinates).

### 2.6. STA-LSTM Framework for Paediatric Pain Detection

Pain classification was performed using the previously published Spatial-Temporal Attention Long Short-Term Memory (STA-LSTM) framework. The input to the network consisted exclusively of the normalised facial landmark trajectories extracted from one-second video clips. No handcrafted facial descriptors or raw image data were used during model training or inference. The STA-LSTM framework combines Long Short-Term Memory (LSTM) layers with spatial-temporal attention mechanisms to learn dynamic facial behaviour from sequential landmark data. LSTM layers captured temporal dependencies across consecutive frames, while spatial-temporal attention mechanisms adaptively emphasised informative landmarks and time segments during feature learning.

Model parameters were initialised through transfer learning from a previously developed adult STA-LSTM model. The final model input consists of 2D facial landmark trajectories derived from 3D-normalised facial landmarks. The model used an input dimension of 956, representing the normalised x and y coordinates at each time point, and a sequence length of 30, corresponding to 30 video frames per second. The LSTM hidden dimension was set to 64, while the output dimension was set to 2, representing the pain and no-pain classes. The model was trained for 60 epochs using binary cross-entropy loss and the Adam optimiser, with a learning rate of 0.001, a weight decay of 10^−6^, and a batch size of 1000. Training and validation datasets were partitioned at the participant level to prevent subject-level leakage; clips from a given child were assigned to either training or validation, never both. Class balancing was applied only to the training dataset, whereas the independent validation dataset retained the original class distribution.

**Figure 2:**
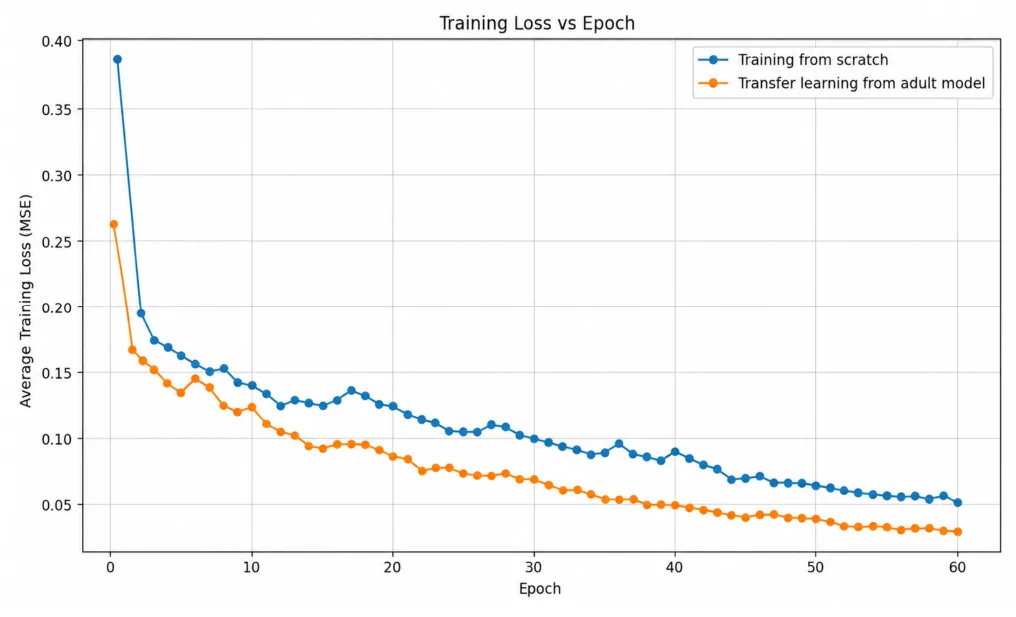
Training Loss of STA-LSTM: Transfer Learning vs. Training from Scratch.

To assess the contribution of transfer learning, an additional control experiment was performed using the same STA-LSTM architecture trained exclusively on the paediatric dataset with random weight initialisation. The same data partitioning, preprocessing procedures, model architecture, and training configuration were used to ensure a direct comparison with the transfer-learning approach. As shown in Figure 2, the transfer-learning model demonstrated more favourable convergence, with a lower initial training loss (approximately 0.26 compared with 0.39 for training from scratch) and consistently lower training loss throughout the 60 epochs. At the end of training, the loss decreased to approximately 0.03 for the transfer-learning model, compared with approximately 0.05 for the randomly initialised model. Both models exhibited stable convergence; however, the consistently lower loss observed for the transfer-learning model suggests that knowledge transferred from the adult STA-LSTM provided a more favourable initialisation for optimisation on the paediatric dataset.

### 2.7. Statistical Analysis

Patient characteristics are presented as counts (percentages), mean ± standard deviation (SD), or median (interquartile range [IQR]), as appropriate. Model performance was evaluated using accuracy, precision, recall, F1-score, and the area under the receiver operating characteristic curve (AUC).

Definitions of performance metrics are summarised in Table 1.

To account for correlation among multiple windows from the same participant, 95% confidence intervals for classification performance were estimated using participant-level cluster bootstrapping. In each of 2000 bootstrap resamples, participants in the held-out test set were sampled with replacement and all their associated windows were retained. Percentile-based 95% confidence intervals were calculated using the 2.5th and 97.5th percentiles of the resulting metric distributions.

## 3. Results

### 3.1. Study Cohort and Dataset Characteristics

A total of 125 children were included in the study. The mean age was 9.1 ± 2.5 years, 92.0% were male, and 96.0% were ASA I. LASER circumcision accounted for 78.4% of procedures. Baseline pain scores were low (median preoperative Numerical Rating Scale (NRS) 0), and the mean Child Pain Anxiety Symptoms Scale (CPASS) score was 1.6 ± 1.6. Postoperative rescue analgesia consisted mainly of paracetamol (25.6%) and ibuprofen (24.8%); opioids were rarely used. Intravenous fentanyl was administered to 1 child (0.8%).

Demographic and clinical characteristics are presented in Table 2.

Continuous video recordings yielded 971 annotated clips of various durations, comprising 161 pain clips (16.6%) and 810 no-pain clips (83.4%). After participant-level partitioning and stride-based balancing, the training set contained 6438 one-second landmark trajectories (3205 pain and 3233 no-pain). The independent validation dataset retained the original class distribution, as follows: 1932 clips; 322 pain (16.7%), 1610 no-pain (83.3%).

### 3.2. Landmark-Based Pain Classification

**Figure 3:**
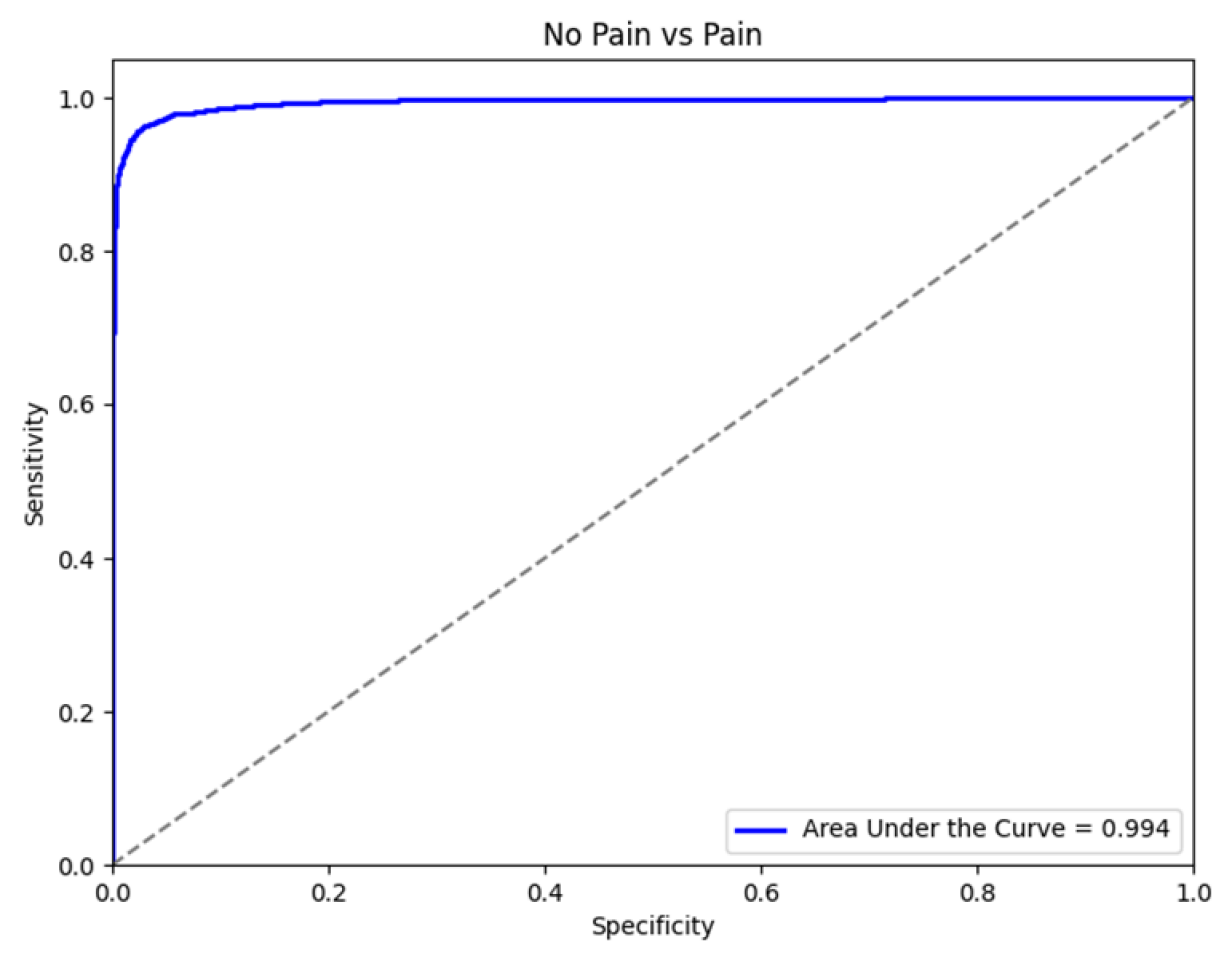
The area under the Receiver Operating Characteristic (ROC) curve for pain vs. no pain.

On the independent validation dataset, receiver operating characteristic (ROC) analysis yielded an area under the curve (AUC) of 0.994 (95% participant-cluster bootstrap CI: 0.980–0.999; Figure 3), indicating excellent discrimination between pain and no-pain trajectories. The ROC curve and AUC were calculated using the continuous model output score for the pain class. Participants included in the validation dataset were independent of those used for model training, with all trajectories from each participant assigned exclusively to one dataset to prevent participant-level data leakage.

For classification, each one-second trajectory was assigned to the class with the higher model output score (argmax); no classification threshold was optimised using the vali-dation dataset. The STA-LSTM correctly classified 315 pain trajectories (true positives) and 1562 no-pain trajectories (true negatives), with 48 false positives and 7 false negatives. This corresponded to an accuracy of 97.15%, sensitivity of 97.83%, specificity of 97.02%, precision of 86.78%, and F1-score of 91.97%. Participant-cluster bootstrap 95% confidence intervals for these performance metrics are presented in Table 3.

The trained network was integrated into a prototype desktop application that processed frontal facial video and generated continuous pain probability estimates during standardised activities (sitting, sit-to-stand transition, standing, and walking), illustrating the feasibility of near-real-time deployment. (Appendix A).

Computational performance was evaluated on a workstation equipped with an Intel Xeon Gold 6230 CPU and 64 GB RAM, with all processing performed on the CPU without GPU acceleration to assess feasibility on broadly accessible computing hardware. The STA-LSTM achieved a mean inference time of 72.0 ± 12.3 ms per 30-frame sequence, with a median of 69.1 ms. The complete processing pipeline, including video frame acquisition, MediaPipe facial landmark extraction, facial landmark normalisation, feature preparation, STA-LSTM inference, and visualisation, achieved a mean end-to-end processing time of 130.2 ms per frame, corresponding to an effective throughput of approximately 7.68 frames per second. These results demonstrate near-real-time operation using CPU-only processing. Once the required 30-frame temporal sequence is available, the relatively low computational latency supports the potential deployment of the framework on general-purpose computing hardware without dedicated GPU acceleration.

### 3.3. Agreement of Reference Pain Annotations

**Figure 4:**
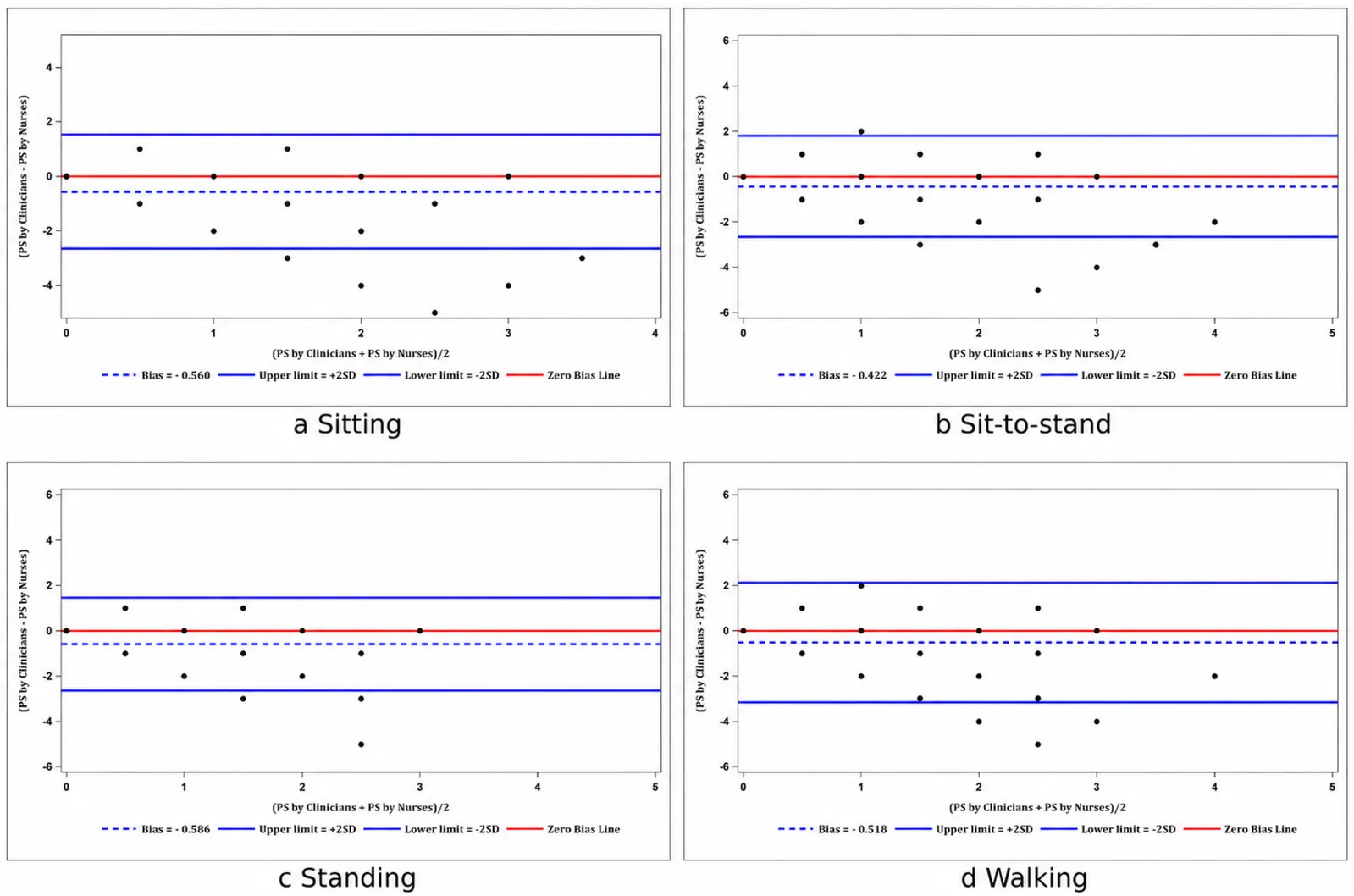
Bland–Altman plot comparing clinician versus nurse pain scores in different scenarios: (**a**) sitting, (**b**) sit-to-stand, (**c**) standing, and (**d**) walking. Black bullets represent paired observations; dashed blue lines show mean bias, solid blue lines show the 95% limits of agreement, and red lines indicate zero difference.

Bland–Altman analysis comparing clinician- and nurse-assigned r-FLACC scores showed mean bias between −0.42 to −0.59 pain score units across the four functional activities, with most observations within the 95% limits of agreement. (Figure 4). Bias was smallest during sitting (Figure 4a) and largest during walking (Figure 4d).

### 3.4. Behavioural Pain Scores Across Functional Activities

Behavioural pain scores for the four standardised activities are summarised in Table 4. Median postoperative pain scores were low across all activities and observer groups (0 to 1). Nurses tended to assign slightly higher scores than clinicians (1 vs. 0), while blinded video reviewers often reported scores of 0, particularly for dynamic activities.

### 3.5. Association Between CPASS and Observer Pain Scores

**Figure 5:**
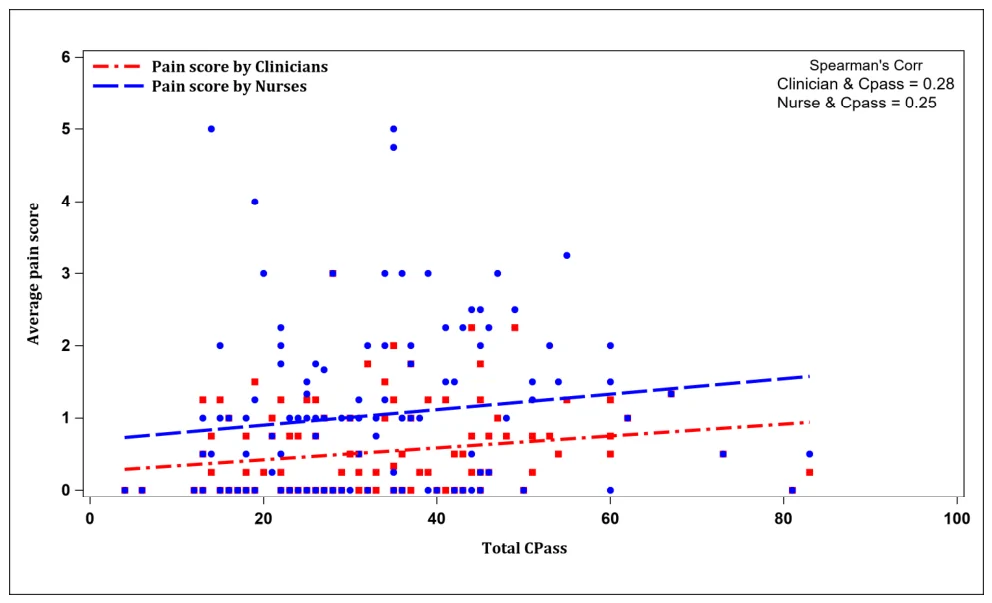
Scatter plot of mean nurse and clinician assigned pain scores plotted against CPASS scores across all observations. Red bullets represent clinician-assigned pain scores, and blue represent nurse-assigned pain scores. The red dash-dotted and blue dashed lines represent the respective fitted trend lines.

Associations between baseline CPASS scores and postoperative observer-assigned pain scores are shown in Figure 5. Spearman correlation coefficients were ρ = 0.28 for clinician-assigned pain scores and ρ = 0.25 for nurse-assigned pain scores.

## 4. Discussion

This study evaluated the applicability of a previously developed STA-LSTM framework for automated paediatric pain detection using two-dimensional facial landmark trajectories derived after three-dimensional geometric normalisation of frontal facial video recordings. Without introducing a new network architecture, we demonstrated that a landmark-based temporal learning framework originally trained on adult data can be adapted to paediatric perioperative settings via transfer learning [12], achieving an accuracy of 97.15%, an F1-score of 91.97%, and an AUC of 0.994 for binary pain classification.

The key contribution of this work is the landmark-based sensing representation and processing pipeline. Rather than analysing facial appearance with image-based models, the system represents facial behaviour as normalised three-dimensional landmark trajectories. This geometric signal reduces input dimensionality, mitigates sensitivity to lighting and background, while preserving facial motion relevant to behavioural pain assessment. Because model inference is performed on landmark coordinates rather than facial images, the approach also reduces reliance on identifiable facial texture, which is advantageous for privacy-conscious clinical deployment [9,10,11,12]. Combined with spatial-temporal attention, the STA-LSTM network learns dynamic patterns of facial movement associated with observer-labelled pain states using only landmark coordinates.

Model evaluation was performed in a perioperative cohort with predominantly low postoperative pain intensity and an imbalance between pain versus no-pain clips. Class balancing was limited to only the training dataset, whereas the validation dataset preserved the original proportions, providing an evaluation that retained the observed class imbalance in this dataset. Under these constraints, the sensing system maintained excellent discrimination, suggesting that, within this dataset, landmark-based temporal features were associated with the observer-derived pain/no-pain reference labels. However, the performance reported in this study should not be assumed to generalise to children undergoing major surgical procedures or experiencing greater pain severity without further validation [4,5,8]. Although the model performed well under these conditions, alternative approaches such as class-weighted loss, focal loss, and oversampling alone were not evaluated. Further studies are needed to determine whether performance remains consistent with different class-imbalance strategies.

Preoperative recordings reflected baseline behaviour, whereas postoperative recordings were obtained during early PACU recovery, when facial expression may also be influenced by residual anesthetic effects, fatigue, anxiety, distress, sedation, or emergence behaviour. Although baseline pain-related anxiety was low (CPASS 1.6 ± 1.6; Table 2) and correlated only weakly with postoperative pain ratings (ρ = 0.25–0.28; Figure 5), these potentially confounding postoperative states were not assessed independently. This distinction is particularly relevant given the low postoperative pain scores across activities and observer groups (Table 3). Furthermore, reference labels were derived from the full r-FLACC score, which incorporates several behavioural domains, whereas the model analysed facial landmarks alone. The detected facial trajectories may therefore represent a broader behavioural response to postoperative discomfort or distress rather than pain-specific facial expression alone [19].

The high AUC of 0.994 should be interpreted within the study context. Sikka et al. similarly reported AUC values of 0.84–0.94 using structured postoperative recordings and binary pain classification in children [20]. In contrast, Fang et al. reported lower performance in a larger and more heterogeneous clinical dataset using four pain-severity categories [6]. Our standardised acquisition protocol, binary classification design, relatively homogeneous cohort, and narrow range of predominantly low pain scores may have reduced the complexity of the classification task. The model’s ability to distinguish different pain intensities or perform under less controlled conditions therefore remains uncertain.

The successful transfer of the adult STA-LSTM framework to paediatric data indicates that the learned temporal representation of facial landmark trajectories is flexible across age groups, despite facial morphological and behavioural differences between adults and children. The existing network architecture was applied without modification following retraining using paediatric landmark sequences. This supports the broader use of landmark-based sensing and temporal attention in the paediatric perioperative setting without redesigning the network architecture [12].

Facial landmark trajectories could be integrated with physiological signals from bedside monitors or wearable sensors, including heart rate variability, respiratory rate, oxygen saturation, electrodermal activity, temperature, and movement [21,22,23,24]. In children, this may improve pain detection when facial expression is weak, intermittently visible, or difficult to interpret because of age, developmental status, sedation, or emergence behaviour. A multimodal system could align these signals in time and combine modality-specific features with the STA-LSTM output through feature-level or decision-level fusion.

Remote photoplethysmography may further allow heart rate and respiratory information to be extracted from the same video stream used for landmark tracking, enabling contact-free multimodal sensing without additional hardware [25]. However, paediatric physiological responses are influenced by fear, agitation, medication, fever, and perioperative autonomic changes and are therefore not specific to pain. Multimodal models should incorporate signal-quality assessment, missing-modality handling, and confidence estimation, and should be evaluated for improved robustness, calibration, and false-alarm reduction rather than as direct physiological measures of pain.

Several limitations merit consideration. The single-centre study design and predominance of minor elective procedures associated with low postoperative pain scores may limit generalisability. Reference labels were derived from observer-rated r-FLACC assessments rather than an absolute physiological gold standard of pain measurement, so model performance reflects agreement with expert observers rather than direct pain intensity [17,26,27]. Facial landmarks may also encode non-pain states such as anxiety, distress or fatigue, which the network could misinterpret as pain. Although we estimated confidence intervals using participant-level cluster bootstrapping to account for within-child correlation among repeated windows, the model was evaluated in only one single-centre held-out test set. External validation at both participant and site levels is required. Furthermore, model evaluation was conducted offline and not integrated into the routine clinical workflow; latency, usability, and acceptance remain to be studied.

The limited diversity of the cohort should also be considered when interpreting the findings. Most participants were male (92.0%) and Malay (83.2%), and circumcision accounted for 78.4% of procedures. The small size of the remaining subgroups did not permit reliable performance analyses by sex, age, ethnicity, or procedure type. Future studies should validate and evaluate the system in multicentre cohorts with more diverse surgical procedures and pain severities to establish the generalisability of the findings, and follow updated reporting guidance for artificial intelligence prediction models, including TRIPOD+AI [28]. Integrating landmark-based facial trajectories with complementary sensing modalities—such as physiological signals and body movement—may further improve robustness in scenarios where facial behaviour alone is insufficient [29,30]. Prospective implementation studies are needed to evaluate system performance, scalability, and clinical impact during real-time use.

## 5. Conclusions

This study demonstrates that a previously developed STA-LSTM framework, using two-dimensional facial landmark trajectories derived after three-dimensional geometric normalisation, can classify observer-labelled pain versus no-pain states in children undergoing elective surgery. The vision-based sensing system achieved high accuracy and discrimination for classifying observer-labelled pain versus no-pain states. During model inference, the system used facial landmark coordinates rather than raw facial-image pixels, reducing reliance on facial texture. These findings support further evaluation of facial landmark trajectories as a practical, potentially privacy conscious, non-contact signal representation for automated behavioural pain assessment in children and provide a basis for further evaluating integrated, multimodal sensor systems in larger and more diverse paediatric populations.

## Figures and Tables

**Table 1:** Performance Matrix and Description [18].

Performance Metrics	Description
True positive (TP)	The instance of correct prediction(s) as a positive class
True negative (TN)	The instance of correct prediction(s) as a negative class
False positive (FP)	The instance of incorrect prediction(s) as a positive class, with the actual class being negative
False negative (FN)	The instance of incorrect prediction(s) as a negative class, with the actual class being positive
Accuracy	The proportion of correctly predicted instance(s) (TP + TN) out of all instances (TP + TN + FP + FN)
Precision	The proportion of the instance(s) of correct prediction(s) as a positive class (TP) out of all predicted positive instances (TP + FP)
Recall	The proportion of the instance(s) of correct prediction(s) as a positive class (TP) out of all actual positive instances (TP + FN)
F1	A harmonic mean of precision and recall, and is calculated as 2 × precision x recall/(precision + recall). An F1 score of 1 indicates its best in quality classification whereas 0 refers to the worst

**Table 2:** Demographic and clinical characteristics of the study cohort.

Parameter	Overall (*n* = 125)
Age (years)	9.1 (2.5)
Male Gender	115 (92.0%)
Race	
Chinese	11 (8.8%)
Malay	104 (83.2%)
Indian	4 (3.2%)
Others ^a^	6 (4.8%)
Weight, kg	35.97 (19.8)
Height, cm	133.2 (19.3)
ASA status	
I	120 (96.0%)
II	5 (4.0%)
Type of surgery	
LASER circumcision	98 (78.4%)
Arthroscopic knee surgery	4 (3.2%)
Tonsillectomy	14 (11.2%)
Removal of implants	1 (0.8%)
Examination of ears/nose	3 (2.4%)
Surgical fixation of fracture	4 (3.2%)
Video assisted thoracoscopy	1 (0.8%)
Pre-surgical/procedural pain score (at rest)	0 (0 [0–10])
Pre-surgical/procedural CPASS	1.6 (1.6 [0–5])
Post-surgical/procedural pain score (at rest)	2.5 (0–3 [0–10])
Rescue Analgesia	
Morphine	0 (0%)
Fentanyl	1 (0.8%)
Paracetamol	32 (25.6%)
Ibuprofen	31 (24.8%)

Values are presented as mean (SD), median (IQR [range]), or number (percentage) as appropriate. ASA, American Society of Anaesthesiologists; CPASS, Child Pain Anxiety and Symptoms Scale; IQR, interquartile range; LASER, Light Amplification by Stimulated Emission of Radiation; SD, standard deviation. ^a^ Others included Filipino (*n* = 3), Pakistani (*n* = 1), and Burmese (*n* = 2).

**Table 3:** Classification performance on the independent validation dataset with participant-cluster bootstrap 95% confidence intervals.

Metric	Performance	95% Cluster-Bootstrap CI
AUC	0.994	0.980–0.999
Accuracy	97.15%	95.5–98.5%
Sensitivity (Recall)	97.83%	95.2–99.5%
Specificity	97.02%	95.1–98.5%
Precision	86.78%	82.3–91.1%
F1-score	91.97%	88.1–95.1%

**Table 4:** Comparison of pain scores assigned by nurses, clinicians, and pain link nurses during postoperative standardised scenarios.

Post-Operative Scenario	Nurses	Clinician	Video Review
Scenario 1: Sitting	0 (0–1 [0–1])	0 (0–1 [0–1])	0 (0–0 [0–0])
Scenario 2: Sit-to-stand	1 (0–2 [0–2])	1 (0–1 [0–1])	0 (0–0 [0–0])
Scenario 3: Standing	1 (0–1 [0–1])	0 (0–1 [0–1])	0 (0–1 [0–1])
Scenario 4: Walking	1 (0–2 [0–2])	1 (0–1 [0–1])	0 (0–1 [0–1])

Values are presented as median (interquartile range (IQR) [range]).

**Figure A1:**
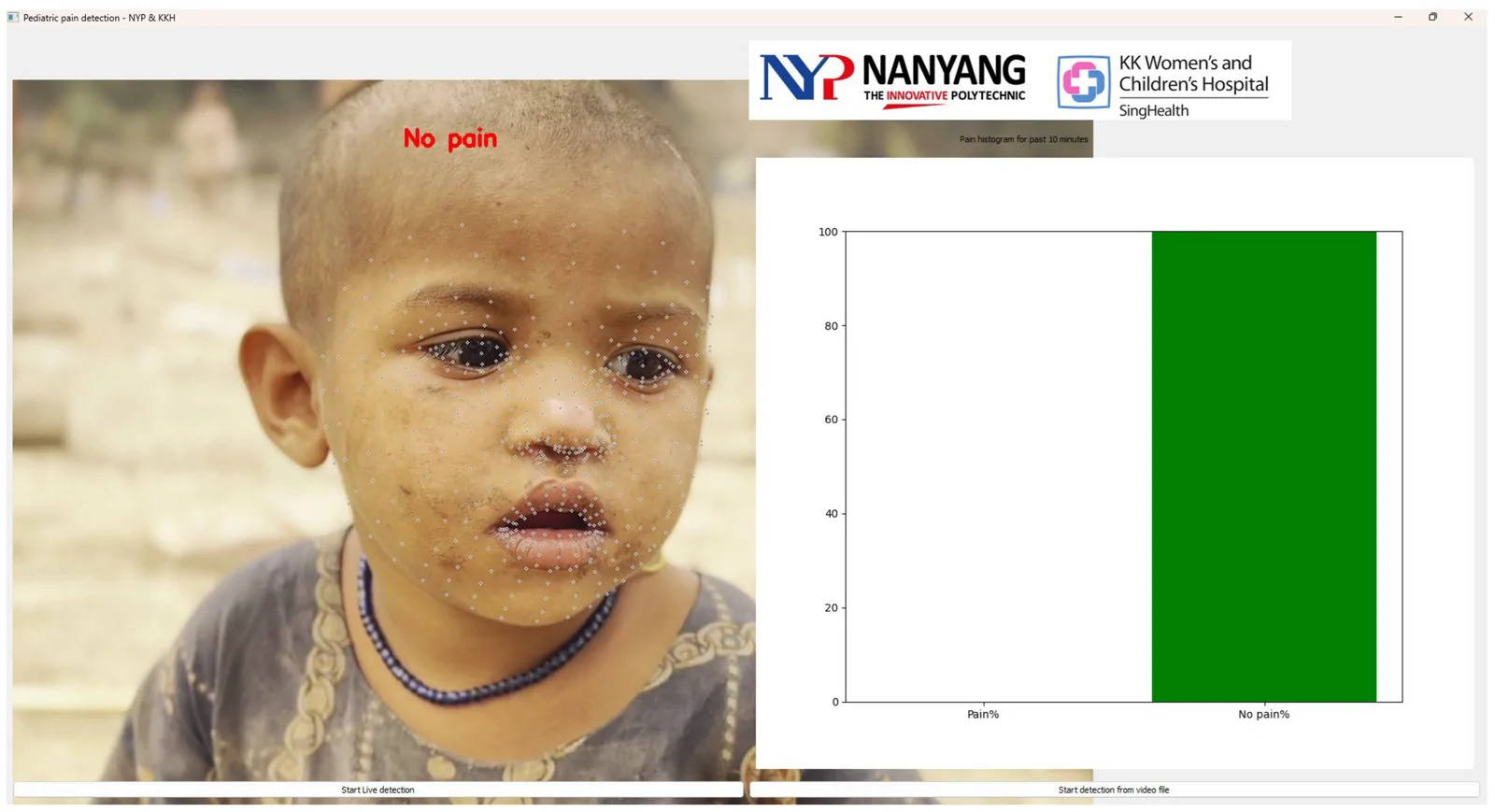
User interface of the custom-developed software for automated pain detection in paediatric patients.

## Data Availability

The data that support the findings of this study are available on request from the corresponding author. The data are not publicly available due to privacy or ethical restrictions.

## Abbreviations

The following abbreviations are used in this manuscript:

AI	Artificial Intelligence
ASA	American Society of Anesthesiologists (physical status)
AUC	Area Under the (Receiver Operating Characteristic) Curve
CPASS	Child Pain Anxiety Symptoms Scale
IQR	Interquartile Range
LASER	Light Amplification by Stimulated Emission of Radiation
LSTM	Long Short-Term Memory
NRS	Numerical Rating Scale
PACU	Post-Anaesthesia Care Unit
r-FLACC	revised Face, Legs, Activity, Cry, Consolability
RGB	Red, Green, Blue
ROC	Receiver Operating Characteristic
SD	Standard Deviation
STA-LSTM	Spatial-Temporal Attention Long Short-Term Memory (Network)

## Appendix A

The developed STA-LSTM model was integrated into a custom-developed PC application (version 1.0; Nanyang Polytechnic, Singapore), providing real-time pain-level distribution and time graphs. The image used in this study was sourced from the Pexels image repository (https://www.pexels.com) and is used in accordance with the Pexels License (https://www.pexels.com/license/), which permits free download and use for academic and publication purposes.
